# Ezrin Regulating the Cytoskeleton Remodeling is Required for Hypoxia-Induced Myofibroblast Proliferation and Migration

**DOI:** 10.3389/fcvm.2015.00010

**Published:** 2015-03-03

**Authors:** Bin Yi, Lin Chen, Jing Zeng, Jian Cui, Guansong Wang, Guisheng Qian, Karine Belguise, Xiaobo Wang, Kaizhi Lu

**Affiliations:** ^1^Department of Anesthesia, Southwest Hospital, Third Military Medical University, Chongqing, China; ^2^LBCMCP, CNRS, Université P. Sabatier Toulouse III, Toulouse, France; ^3^Institute of Respiratory Disease, Xinqiao Hospital, Third Military Medical University, Chongqing, China

**Keywords:** pulmonary myofibroblast, hypoxia, ezrin, cell proliferation, pulmonary vascular remodeling, pulmonary artery smooth muscle-like cells

## Abstract

**Background:** Hypoxia pulmonary arterial hypertension (HPAH) is a disease of the small vessels characterized by sustained vasoconstriction, thickening of arterial walls, vascular remodeling, and progressive increase in pulmonary vascular resistance, thus leading to right heart failure and finally death. Recent evidence demonstrated that massive pulmonary artery smooth muscle-like cells (PASMLCs) accumulating in the intima might also be developed from the differentiation of pulmonary myofibroblast (PMF) of tunica media. And PMF appeared the phenomenon of the cytoskeleton remodeling. So, it would be important in the clarification of the pivotal factors controlling this cytoskeleton structure change.

**Methods:** PMFs were cultured from the normal rats and then divided into three groups and incubated by normal or hypoxic conditions respectively. mRNA level was evaluated by real-time reverse transcription polymerase chain reaction, and protein expression was detected by western blot. Cell proliferation was determined by the MTT and thymidine incorporation assay.

**Results:** Here, we report that the hypoxia increased the expression levels of ezrin mRNA and protein in PMFs, which might explain that the expression of cytoskeletal proteins (destrin, a1-actin, and a1-tubulin) in PMFs was significantly induced by hypoxia. After inhibiting ezrin in PMFs by siRNA transfection, we found the over-expression of cytoskeletal proteins induced by hypoxia was significantly suppressed at all time-points. Additionally, we found that hypoxia or over-expression of ezrin through adenovirus-mediated ezrin gene transfection significantly increases the proliferation and migration of PMFs, and which could be inverted by the transfection of siRNA.

**Conclusion:** These findings suggest that ezrin regulating of aberrant dysregulation of cytoskeletal proteins may be the major cause of PMFs’ proliferation and migration under the condition of hypoxia and may, therefore, play a fundamental role in the accumulation of PASMLCs of HPAH.

## Introduction

Hypoxia pulmonary arterial hypertension (HPAH) is a disease of the small pulmonary arteries characterized by sustained vasoconstriction, thickening of aortic wall, vascular remodeling, and progressive increase in pulmonary vascular resistance, thus leading to right heart failure and finally death (1–3). It has been proven that the massive accumulation of pulmonary artery smooth muscle-like cells (PASMLCs) in the vascular intima is likely the major reason resulting in arterial remodeling and vascular lumen narrowing, and thus it is the main cause of cor pulmonale in chronic obstructive pulmonary disease (COPD) (4). Although some early studies suggested that this massive cell accumulation in the intima just stemmed from the proliferation and migration of PASMCs in tunica media, recent evidence demonstrated that massive PASMLCs accumulating in the intima might also be developed from the differentiation of pulmonary myofibroblasts (PMFs) of tunica media (5).

Myofibroblasts (MFs) were first described in experimental wound healing by observation method of electron microscopy, as fibroblastic cells located within granulation tissue and exhibiting bundles of microfilaments (6). Since then, evidence from many laboratories has indicated that MFs play a pivotal role in tissue repair and remodeling; moreover, they are a key player in different pathological conditions such as hypertrophic scars, fibromatosis, systemic sclerosis, organ fibrosis, and stroma reaction to epithelial tumors (7–11). In all forms of pulmonary hypertension, the early appearance of adventitial PMFs is an event that precedes intimal and medial changes. Here, an important factor leading to pulmonary hypertension is hypoxia, which is recognized as a critical factor in vascular remodeling. During hypoxia-mediated pulmonary hypertension, hypoxia can induce fibroblast to myofibroblast (F-MF) transition, and also stimulate the production of growth factors and cytokines, such as transforming growth factor-β, thrombin, endothelin (12, 13). In addition, as a major downstream of hypoxia effect, adventitial cells expressing mesenchymal stem cell markers could be a source of MFs and contribute to arterial remodeling (14). Taken together, the role of MFs in the pathogenesis of HPAH has thus drawn more and more attention by researchers. However, the molecular mechanism of hypoxia-induced MF differentiation for PASMLCs is unclear and thus demands further study.

Because hypoxia-induced pulmonary vessel remodeling is often associated with the remodeling of cytoskeleton structure, it will be not only interesting but also important in the clarification of the pivotal factors controlling this cytoskeleton structure change. Ezrin, a cellular protein initially isolated as a cytoskeletal component of intestinal microvilli and a substrate for tyrosine kinase, shows 77, 73, and 43% homology with three other proteins, radixin, moesin, and merlin encoded by neurofibromatosis type II (NF2) tumor suppressor gene. These molecules comprise the ezrin/radixin/moesin (ERM) family. ERM proteins, a group of band 4.1 superfamily, act as linker proteins that interact with many cytoskeleton such as a1-actin, a1-tubulin, F1-action, and destrin. However, the absolute different properties of ezrin and merlin could influence cell proliferation and function (15–18). Ezrin is the essential molecule that stimulates proliferation and migration, whereas merlin is the molecule that inhibits cell proliferation of many cancer cells from PASMCs, pulmonary microvascular endothelial cells (PMVECs), and fibroblast, all of which are involved in the hypoxia-induced pulmonary vessel remodeling at the same time (19, 20). Therefore, it has been proposed that ezrin might be probably involved in the different regulatory processes in hypoxia-induced PASMLCs proliferation and migration. However, there is no definitive evidence for this assertion.

In the present study, we demonstrate that ezrin protein mediates proliferation and migration of MFs via regulating their cytoskeletal remodeling. We found that over-expression or silence of ezrin protein increase or decrease the expression of cytoskeletal proteins in human MF respectively. More importantly, as our hypothesis that MFs might be also involved in the cell proliferation and migration induced by hypoxia, we observed that hypoxia can strongly induce the expression of ezrin, which mediates the hypoxia-induced growth and migration of MFs as well as the expression control of several cytoskeleton proteins involved in vessel remodeling.

## Materials and Methods

### Reagents

Rat MF line, built in our lab and used in our initial studies were cultured in low glucose Dulbecco’s modified Eagle’s medium (DMEM) (PAA, USA) supplied with 10% fetal calf serum (FCS) (HyClone, USA), as previously described (21). A rabbit polyclonal anti-ezrin antibody, a rabbit polyclonal anti-MHC antibody, a rabbit polyclonal anti-calponin antibody, a rabbit polyclonal anti-ezrin antibody were bought from NeoMarkers company of USA.

### Adenovirus transduction

Inoculated PMFs of a similar concentration (10^6^/cm^2^) were divided into a transduced group, a non-transduced group, and an empty vector group. Each group had the following treatments: normoxia (*T*_0_), 24 h (*T*_1_), and 48 h (*T*_2_) treatment with 3% O_2._ When these cells were 80% confluent, the original serum was replaced with 0.1% FCS.

The recombinant adenovirus (Ad) that carried the full-length cDNA of the ezrin gene and green fluorescent protein (GFP) was constructed in our previous study. After 24 h of synchronous growth, the PASMCs received the hypoxia treatment. In addition, 24 h prior to treatment, adenovirus-mediated ezrin gene (Ad-ezrin) with a multiplicity of transduction (MOI) of 60 was added to the medium of the Ad-ezrin-transduced group. Subsequently, these cells were placed upside-down under an immunofluorescence microscope to determine transduction efficiency.

### Immunofluorescence microscopy

Pulmonary myofibroblasts were plated at concentration of 2 × 10^5^ cells/ml on glass coverslips and allowed to be adherent overnight. Coverslips were then washed with PBS and fixed in 4% paraformaldehyde for 15 min. After being permeabilized in 0.5% Triton X-100 for 15 min at room temperature, cells were washed by PBS and incubated at 4°C overnight in PBS (PH 7.0) containing 1% bovine serum album with rabbit anti-human ezrin antibody or control 10% goat serum (Biostar, Wuhan, China). After that, cells were washed three times by PBS and incubated at 4°C for 1 h with antirabbit IgG conjugated to TRITC (Biostar). Nuclei were stained by 4′,6-diamidino-2-phenylindole (DAPI). After the final wash, cells were mounted and then visualized using an Olympus fluorescent microscope.

### Real-time RT-PCR analysis

Total RNA was processed using Tripure kits to perform the reverse transcription (RT)-polymerase chain reaction (PCR) experiments. RT was performed with 2 μg RNA, Oligo (dT) 15 (Sangon, China), dNTP (Sangon, China), and the reaction buffer supplied with M-MLV reverse transcriptase (Promega, USA). The fluorescence intensity ratio of the target gene to GAPDH was used as a measure of the relative gene expression. The PCR amplification reaction conditions were as follows: 94°C 1 min, 59°C 30 s, 72°C 1 min in 32 cycles. Specific primer sequences were used for the amplification of ezrin (forward primer: 5′-GTGGGATGCTCAAAGATAATGC-3′; reverse primer: 5′-CACCTCGATGGTGTCAGGCT-3′; 375 bp) (22), and GAPDH (forward primer: 5′-TGGAGTCCACTGGCGTCTTCAC-3′; reverse primers: 5′-CATGAGGTCCACCAGCCCTGTTG-3′; 698 bp). After amplification, 10 μl of PCR product was used for electrophoresis on a 1.5% agarose gel, whose result was scanned by an Alpha Imager (set as a gray scale). The experiment was performed independently four times.

### Western blot

Cells were harvested under the described conditions and lysed in a RIPA buffer (0.5% Non-idet P-40, 10 mM Tris, pH 7.4, 150 mM NaCl, 1 mM EDTA, 1 mM Na_3_VO_4_) with a protease inhibitor (1 mM PMSF). The BCA protein assay was used to quantify protein. Briefly, after Mix reagents, sample were added and incubated for 30 min and read at 562 nm. Equal quantities of protein were loaded for sodium dodecyl sulfate-polyacrylamide gel electrophoresis (SDS-PAGE). Following transfer to PVDF membranes, samples were incubated with primary antibody (1:1000 dilution) at 37°C for 1 h or overnight at 4°C. The membrane was washed twice with TBS, and the bound antibody was detected using an IgG-HRP secondary antibody (1:500 dilution) for 30 min. Blots were detected using an enhanced chemiluminescence system (Amersham, UK). Another membrane prepared by the same protocol was probed with anti-GADPH antibody (Santa Cruz Biotechnology, Santa Cruz, CA, USA) to normalize sample loading.

### RNA interference

siRNA was used to knock down ezrin in the human PASMCs. Non-silenced and siControl human PASMCs were used as control. The sequence of siRNAs were siRNA-ezrin 1 (5′-GGACACUUGGAUUUUUUUUtt-3′), siRNA-ezrin 2 (5′-GGUGGUAAAGACUAUCGGCtt-3′), siRNA-ezrin 3 (5′-GGAUUUCCUACCUGGCUGtt-3′) (22). An electroporation instrument (Bio-Rad, Hercules, USA) was employed for transfection. Briefly, 400 μl of human PASMCs (2.5 × 10^6^ cells/ml cold EBM-2) together with 1.4 μg siRNA were added into a prechilled electrode gap cuvette (Bio-Rad). Then, the cuvette was inserted into an electrotransfer channel. After cells were shocked once using square wave 250 V for 1 min, the cuvette was taken out immediately and 0.5 ml of complete medium EGM-2 pre-warmed at 37°C was added. Finally, cells were seeded into six-well culture plates and incubated at 37°C in a humidified 5% CO_2_ chamber.

### PMFs migration assay

Migration assays were performed using the scratch wound motility assay. MFs were seeded in six-well plates (1.50 × 10^5^ cells/well) and grown to confluence. Twenty-four hours after serum deprivation, cells from each group were mounted on a reusable template to create a standard wound (<3 mm). Wound closure rate was followed with a reference point in the field of the wound at the bottom of the plate by direct microscopic visualization. The procedure permitted the imaging of the identical spot each time. The remaining cell-free area was determined via microphotography that was performed 24 h after injury.

### Tritiated thymidine incorporation assay

Cells with 2 × 10^4^/ml were inoculated in a 96-well culture plate. Each phase had three wells that were cultured for 2 days before hypoxia treatment. One microcurie tritiated thymidine (^3^H-TdR; 1 μCi/well) was added to each well 6 h prior to the end of the culturing phase. Subsequently, the culture was stopped at the proper phase, and the medium was removed. The incorporation was stopped with cold PBS solution. The cells were digested with 0.25% trypsin and collected on a glass fiber filter with a multi-head harvester. Cells were washed three times with physiological saline stabilized with 10% trichloroacetic acid, destained with absolute ethanol, dried for 30 min at 80°C, transferred into scintillation fluid, and counted in a liquid scintillation counter (counts/min, cpm).

### MTT assay

Cells between 10^3^ and 10^4^ were inoculated into each well in a 96-well plate. The plate was placed in 5% CO_2_ and cultured for 24 h. Each group received the appropriate treatment for its specific experimental condition. The plate was cultured for an additional 24 h after replacing the medium with a serum-free medium. Next, 20 μl MTT solution (5 mg/ml) was added, and the plate was inoculated for 4 h at 37°C. The supernatant from each well was then carefully discarded. Finally, 150 μl DMSO was added to each well, and the crystals were fully dissolved using 10 min oscillations. The absorbance value of each well was measured using an ELISA reader with 490 nm as the test wave length and 280 nm as the reference wave length.

### Statistical analysis

All data are represented as means ± SEM. Comparisons between groups were performed using a Student’s *t*-test or a Mann–Whitney *U*-test for parametric or non-parametric tests, respectively. The results were considered statistically significant when *p* < 0.05.

## Results

### Hypoxia induced the proliferation and migration of PMFs

**Figure 1:**
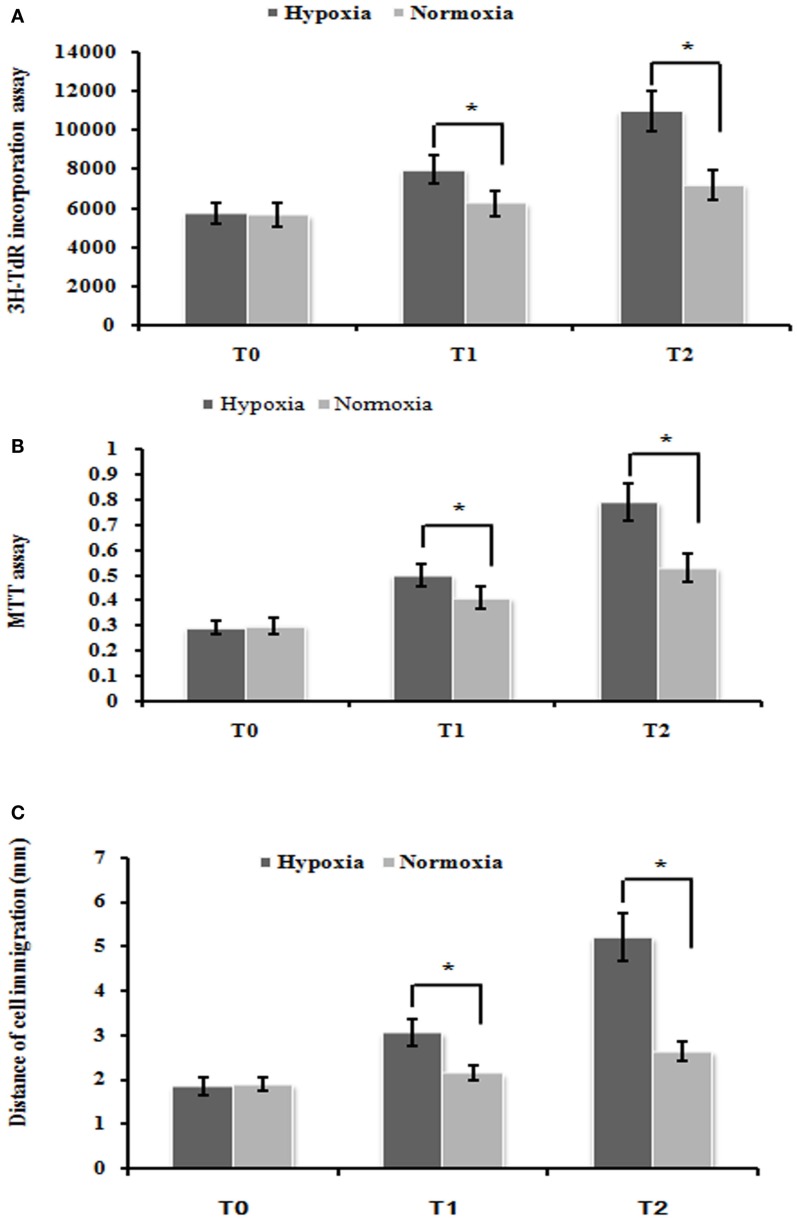
**Hypoxia markedly enhanced PMFs proliferation and migration**. **(A)** DNA synthesis was assessed by a ^3^H-TdR incorporation assay. **(B)** Cell viability was measured using an MTT assay. **(C)** Cell migration ability was assessed by scratch wound motility assay. The distance of cell migration in all groups and time-points is displayed. Each data point represents the mean ± SEM of four independent experiments. *p < 0.05 vs. normoxia condition; *T*_0–2_: MFs were treated with normoxia or hypoxia condition for 0 h (*T*_0_), 24 h (*T*_1_), 48 h (*T*_2_), respectively.

To test the effect of cell proliferation by hypoxia treatment, we used the ^3^H-TdR incorporation assay and MTT assay to measure the rate of DNA synthesis and cell proliferation, respectively. PMFs under hypoxia condition exhibited significantly higher DNA synthesis than those cultured under normoxia condition at each time-point measured (Figure 1A). Similarly, the rate of PMFs proliferation was strongly induced under hypoxia condition compared with the control normoxia status (Figure 1B). In addition, hypoxia prominently enhanced the migrating ability of PMFs (Figure 1C). Taken together, all these results demonstrated that hypoxia treatment positively promoted the proliferation and migratory ability of PMFs.

### Hypoxia enhanced the expression of ezrin gene and cytoskeleton proteins in PMFs

**Figure 2:**
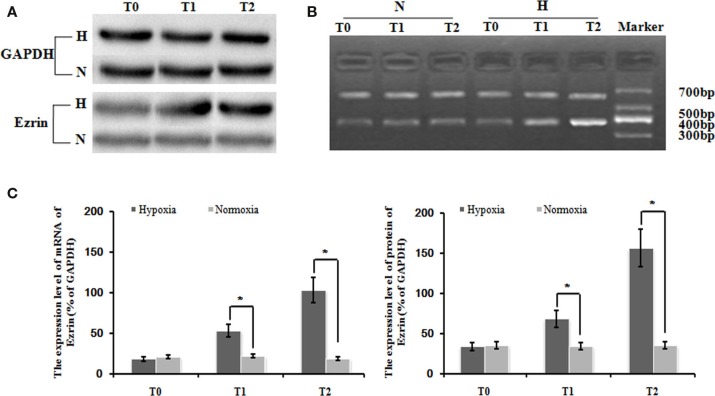
**Levels of ezrin mRNA and protein in PMFs induced by hypoxia at each time-point**. Exposure to hypoxia condition significantly increased expression of the ezrin gene, including the transcript and protein levels in PMFs (*p* < 0.05). **(A)** The electropherogram of western blotting. **(B)** The electropherogram of RT-PCR. **(C)** The density of the expression of mRNA and protein of ezrin compared with GAPDH is displayed. Each data point represents the mean ± SEM of four independent experiments. *p < 0.05 vs. normoxia.

Our following question is which factor(s) might be involved in the control of the induced cell proliferation and migration of PMFs. Here, we focused on some cytoskeleton targets because cytoskeleton protein levels and structure change are often observed during HPAH process. Consistent with our hypothesis, hypoxia treatment significantly increased the gene expression of ezrin, compared with normoxia treatment. The relative expression of ezrin mRNA under hypoxia condition was significantly higher than in that under normoxia condition at different time-points (Figures 2A,B). We also detected the similar induction of ezrin protein expression in PMFs under treatment with hypoxia.

Interestingly and consistently, exposure in hypoxia condition significantly increased the expression of several cytoskeleton proteins such as a1-actin, a1-tubulin, and destrin (Figure 2C). Thus, it indicates that ezrin might be a critical factor that can be induced by hypoxia to result in the strong induction of cytoskeleton structure observed in HPAH disease.

### Ad-ezrin effectively up-regulated ezrin transcription and protein expression in PMFs and siRNA effectively down-regulated ezrin protein expression induced by hypoxia

**Figure 3:**
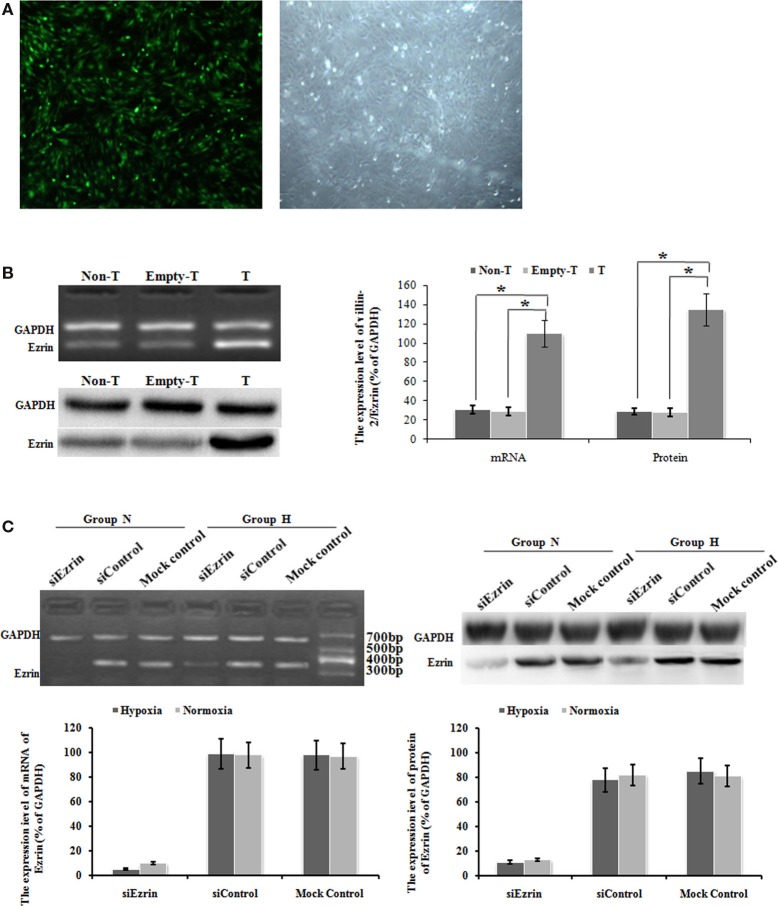
**Ad-ezrin transfected into PMFs was strongly expressed and the protein was found in the PMFs**. And the expression of Ezrin was successfully down-regulated in PMFs by siRNA. **(A)** At 24 h after transfection with Ad-ezrin, PMFs had a normal structure with no observable pathological characteristics (magnification 40×). Bright green fluorescence was observed in nearly all cells using an inverted fluorescence microscope following transfection with Ad-ezrin containing the green fluorescent protein (GFP) gene (magnification 40×). **(B)** The expression of the ezrin mRNA and protein in PMFs 24 h in each group was determined by RT-PCR and western blotting. GAPDH was used as an internal control. **(C)** The expression of ezrin was successfully down-regulated in siRNA. PMFs were incubated with normoxia or hypoxia treated concurrently with siRNA. Ezrin transcription level dramatically diminished after 48 h of transfection compared with levels in the siControl and mock control groups. Following 48 h transfection, the protein expression of ezrin was strongly suppressed by ezrin siRNA. Each data point represents the mean ± SEM of four independent experiments. *p < 0.05; Non-T, non-transfection with Ad-ezrin; empty-T, empty vector transfection; T, transfection with Ad-ezrin; Group N, normoxia condition; Group H, hypoxia condition.

To test whether the respective change of ezrin expression might affect the cell proliferation and migration of PMFs, we introduced either ectopic ezrin-expressing PMFs or ezrin-depleting PMFs. After virus transduction with GFP-tagged ezrin, bright green fluorescence was observed in the cytoplasm and nuclei of more than 95% cells (Figure 3A). Compared with non-transduced or empty vector-transduced control PMFs, in both of which low ezrin transcript and protein expression levels were detected, ezrin-GFP-transduced PMFs showed a significant increase in both transcription and protein levels (Figure 3B).

In the opposite case, we transfected the SiRNA targeting ezrin to knock down the gene and protein expression of endogenous ezrin in PMFs. At 48 h after siEzrin transfection, there was no significant difference in the expression of ezrin between the siControl and the mock control groups, while we observed a remarkable reduction of both ezrin gene and protein levels in siEzrin-expressing PMFs. More importantly, the knockdown of ezrin strongly blocked the respective induction of ezrin by hypoxia treatment (Figure 3C).

### Over-expression of ezrin gene in normoxia condition stimulated the proliferation and migration of PMFs

**Figure 4:**
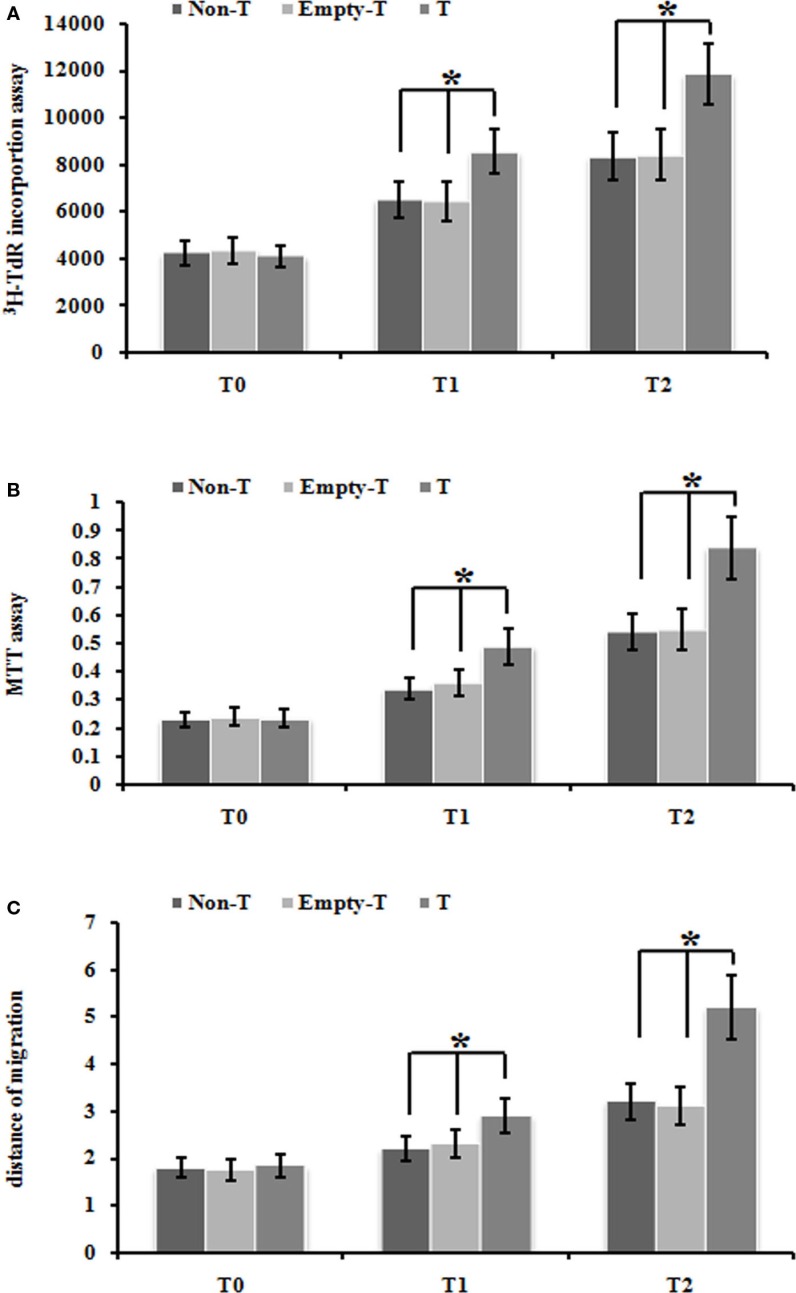
**Over-expression of ezrin protein induced by Ad-ezrin significantly enhanced the proliferation and migration of PMFs, just like hypoxia**. **(A)** DNA synthesis was assessed by a ^3^H-TdR incorporation assay. **(B)** Cell viability was measured using an MTT assay. **(C)** Cell migration ability was assessed by scratch wound motility assay. The distance of cell migration in all groups and time-points is displayed. Each data point represents the mean ± SEM of four independent experiments.*p < 0.05 vs. T; non-T, non-transfection with Ad-ezrin; empty-T, empty vector transfection; T, transfection with Ad-ezrin.

Firstly, we used our ezrin-transduced PMFs tested above (Figures 3A,B) to test whether over-expression of ezrin in PMFs under the normoxia condition could phenotype the cell proliferation and migration ability enhanced by hypoxia treatment. Ezrin-overexpressing PMFs under normoxia condition exhibited the significant increase of both DNA synthesis and cell proliferation levels, compared with group mock-vector and non-transduction PMFs (Figures 4A,B). Moreover, we also detected the prominently enhanced cell migration ability by the over-expression of ezrin in PMFs (Figure 4C). Taken together, all these results indicate that the enhanced levels of ezrin gene and protein expression under hypoxia could account for the respective increase of cell proliferation and migration in HPAH.

### Silencing ezrin protein expression inhibited the proliferation and migration of PASMCs induced by hypoxia or ad-ezrin

**Figure 5:**
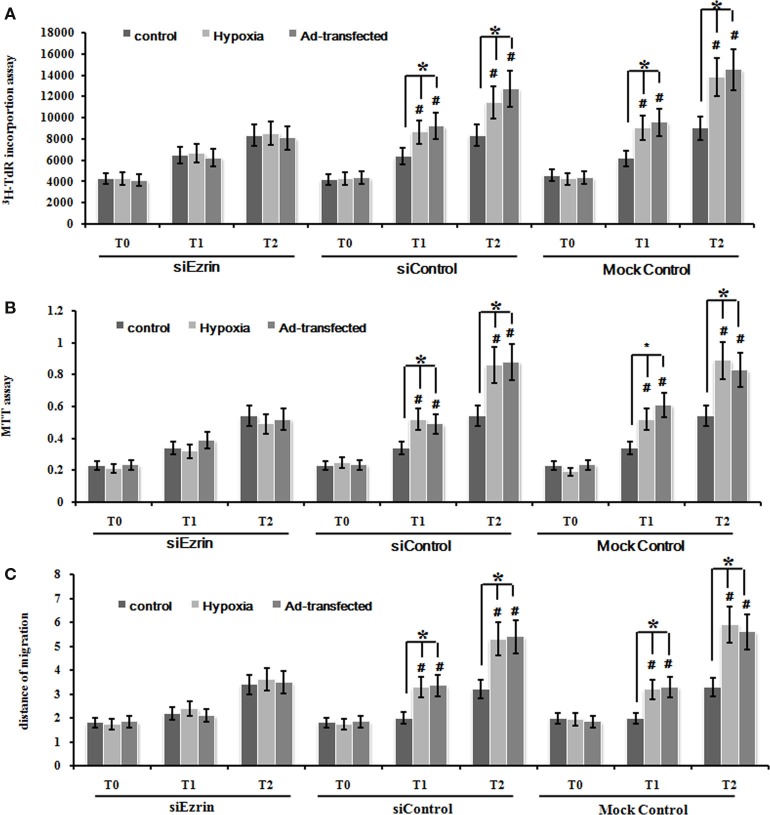
**Silencing ezrin protein expression inhibited the proliferation and migration of PMFs induced by hypoxia or ad-ezrin**. **(A)** DNA synthesis was assessed by a ^3^H-TdR incorporation assay. **(B)** Cell viability was measured using an MTT assay. **(C)** Cell migration ability was assessed by scratch wound motility assay. The distance of cell migration in all groups and time-points is displayed. Each data point represents the mean ± SEM of four independent experiments.*p < 0.05.

To further confirm whether this hypoxia-enhanced expression of ezrin definitely led to the significant increase of cell proliferation and migration ability, we used the ezrin SiRNA-expressing PMFs that we already tested in the knockdown efficiency above (Figure 3C). DNA synthesis, cell proliferation, and migration levels were significantly inhibited in PMFs with ezrin SiRNA expression compared to the siControl or mock control cells (Figures 5A–C).

### Silencing ezrin protein expression inverted the change of cytoskeleton proteins induced by hypoxia

**Figure 6:**
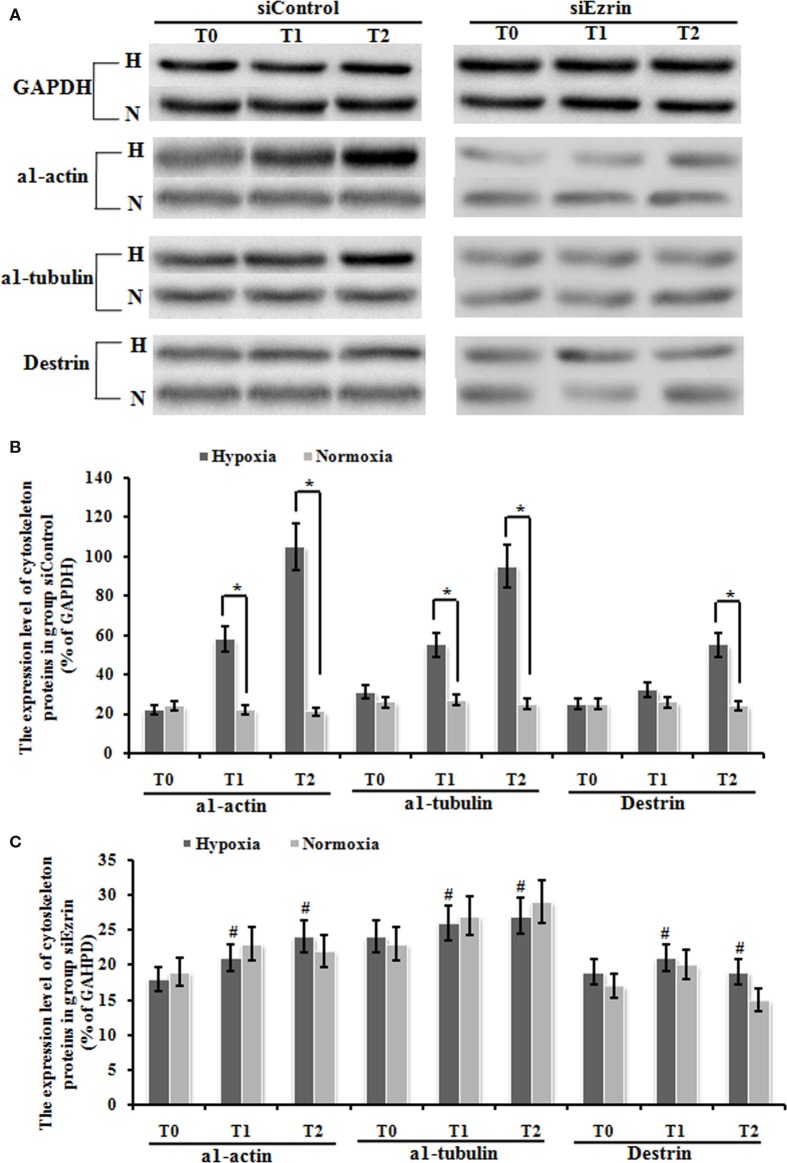
**The expression change of cytoskeleton proteins, including α1-actin, a1-tubulin, and destrin in myofibroblast was up-regulated significantly under hypoxic conditions, and this up-regulation was blocked by the transduction of siEzrin**. **(A)** The electropherogram of the western blot analysis. GAPDH was used as an internal control. **(B)** Quantification of western blots using densitometry in Group siControl. **(C)** Quantification of western blots using densitometry in Group siEzrin. Each data point represents the mean ± SEM of four independent experiments. *p < 0.05 vs. normoxia condition; ^#^*p* < 0.05 vs. group siControl.

We have already shown that exposure under hypoxia condition significantly increased the protein expression of a1-actin, a1-tubulin, and destrin cytoskeleton factors (Figures 6A,B), which indicates that this potential induction of cytoskeleton proteins and structure might be involved in tissue modeling involved in HPAH disease. Our final question is whether these enhanced cytoskeleton protein levels could stem from ezrin which can be prominently induced by hypoxia status. Knockdown of ezrin in PMFs under hypoxia treatment completely inhibited the induction of these three cytoskeleton proteins under hypoxia condition (Figures 6A–C), confirming our hypothesis.

## Discussion

It is currently believed that the accumulation of mass PASMLCs in the vascular intima is likely the major reason leading to arterial remodeling and vascular lumen narrowing. Although some early studies suggested that the accumulation of mass PASMLCs in the intima is just from the migration and proliferation of PASMCs in tunica media, recent evidence showed that mass PASMLCs accumulating in the intima are differentiated from PMF of tunica media too (12). Previous studies have shown that exposure of lung fibroblasts to hypoxia stimulates the fibroblast proliferation and its differentiation into PMFs (8, 12, 23). More importantly, PMFs characteristically synthesize α-SMA, a most commonly used molecular marker, which contributes to stronger contractile activity. Contractile PMFs are responsible for the irreversible alterations of the lung parenchyma that result in accumulation of stiff scar tissue, ultimately leading to deterioration of lung function (13, 23, 24).

In the present study, we firstly demonstrated that the expression of cytoskeletal proteins, including a1-actin, a1-tubulin, and destrin in PMFs is up-regulated significantly under hypoxic condition compared to normoxia status. This suggested that hypoxia promotes the differentiation of PMFs to be a kind of PASMLCs. Combined with previous finding, it indicated that hypoxia might stimulate the transformation of lung fibroblasts into PMFs, and further develop the PMFs into one subgroup of PASMLCs.

Secondly, our work strongly clarified the underlying mechanism for this hypoxia-induced PMFs transformation which has been unclear for a long time. It has been well-known that TGF-β1 induces the PMFs cell transformation and the cytoskeleton proteins synthesis critical for the formation of PASMLCs. Notably, we have previously reported that hepatopulmonary syndrome’s rat serum induces PMVECs to differentiate into PASMLCs, during which the TGF-β1/Smads signal and annexin A1/A2 proteins might play a key role in this differentiation and transformation of PASMLCs (25). However, the molecular mechanism of hypoxia-induced fibroblast-to-PMFs and the cytoskeletal remodeling in it is kept unknown. Here, our study demonstrated that ezrin, a critical cytoskeleton protein, plays critically important role in this process. Our finding is highly consistent with many previous studies that multiple cells including PASMCs, PMVECs, and PMFs, involved in the key pathogenesis process of PVR (12, 23, 26, 27), show the prominent cytoskeleton remodeling when exposure to hypoxia or other stimulators. And it strongly suggested that ezrin functions as the core regulator of cytoskeleton remodeling observed in all these kinds of cells, thus could be used as the new suitable therapeutics target of diseases associated with vascular remodeling.

Ezrin, one of the ezrin–radixin–moesin proteins, is involved in the formation of cell membrane processes such as lamellipodia and filopodia, and thus acts as a membrane–cytoskeleton linker (20). Our results showed that ezrin protein can be significantly positively regulated by exposure to hypoxia, and this ezrin increase accounts for the ability induction of proliferation and migration of PMFs. Our findings suggested that ezrin protein might be a key regulatory protein in the over-expression of cytoskeletal proteins in PMFs and might control not only the hypoxia-induced transformation of fibroblast-to-PMFs, but also the migration and proliferation capability of PMFs. However, it is still unclear about the relationship between the differentiation of PMFs and several signaling pathway of factors such as TGF-β1 and annexin A1/2, and the functional link between all these controlling signals and factors.

In summary, our novel findings provided the evidence supporting the hypothesis that the ezrin-mediated cytoskeletal remodeling strongly controls the MFs proliferation and migration induced by hypoxia. Our future understanding of abnormal cytoskeletal remodeling and the endogenous regulatory machinery of the cytokine signaling cascade will be useful to provide a basis for circumventing the process of vascular remodeling and the creation of targeted therapies for diseases that are associated with vascular remodeling.

## Conflict of Interest Statement

The authors declare that the research was conducted in the absence of any commercial or financial relationships that could be construed as a potential conflict of interest.

## Abbreviations

3H-TdR, tritiated thymidine; Ad, adenovirus; Ad-ezrin, adenovirus-mediated ezrin gene; COPD, chronic obstructive pulmonary disease; ERM, ezrin/radixin/moesin; F-MF, fibroblast to myofibroblast; GFP, green fluorescent protein; HPAH, hypoxia pulmonary arterial hypertension; MFs, myofibroblasts; MOI, multiplicity of transduction; NF2, neurofibromatosis type II; PASMLCs, pulmonary artery smooth muscle-like cells; PMF, pulmonary myofibroblast; PMFs, pulmonary myofibroblasts; PMVECs, pulmonary microvascular endothelial cells.
